# Chagas Disease in Europe

**DOI:** 10.3390/tropicalmed8120513

**Published:** 2023-12-01

**Authors:** Marta Gonzalez-Sanz, Clara Crespillo-Andújar, Sandra Chamorro-Tojeiro, Begoña Monge-Maillo, Jose A. Perez-Molina, Francesca F. Norman

**Affiliations:** 1 National Referral Unit for Tropical Diseases, Infectious Diseases Department, Ramón y Cajal University Hospital, IRYCIS, 28034 Madrid, Spain; 2 CIBER de Enfermedades Infecciosas, Instituto de Salud Carlos III, 28029 Madrid, Spain; 3 Universidad de Alcalá, 28801 Alcalá de Henares, Spain

**Keywords:** *Trypanosoma cruzi*, vertical transmission, migration, neglected tropical disease

## Abstract

Chagas disease is currently present in many non-endemic countries and remains a neglected tropical disease globally. A review of the literature identified significant gaps and scarcity of updated information from European countries, with most studies reporting data from Spain and Italy. The index of underdiagnosis may be as high as 70%, affecting mainly females of child-bearing age. Standardized screening of fertile, non-pregnant, women from endemic countries and subsequent treatment is considered an essential strategy to control transmission and prevent new cases, yet no uniform legislation for screening risk groups exists. There is heterogeneity in Europe in terms of preventive strategies to avoid transfusion-related transmission of Chagas disease, not necessarily in line with the European directives, with some countries conducting systematic screening for *T. cruzi* infection in blood donors, whilst others rely on pre-transfusion questionnaires. The growing burden of the infection in resource-rich areas may provide an opportunity for progress in certain aspects of control and prevention. Options for improving screening strategies, management and linkage to care are reviewed.

## 1. Introduction

Chagas disease (CD), caused by the protozoan *Trypanosoma cruzi*, was previously a geographically restricted infection but may now be considered a global health problem. According to the World Health Organization, an estimated 6 to 7 million people are infected with *T. cruzi* worldwide and an estimated 75 million people are at risk of infection [1].

Vectorial transmission only rarely occurs in non-endemic areas, but other modes of acquisition, such as the vertical route, may lead to transmission of the infection, particularly as many individuals in the chronic phase remain undiagnosed. The indexes of underdiagnosis and undertreatment have been noted to be high in many non-endemic countries (around 70% and 80%, respectively, according to one recent study), especially in females of child-bearing age [2,3,4]. Although a sustained decline in the number of cases has been observed globally and the majority of cases remain concentrated in endemic countries of Latin America, increases have been documented in certain areas, such as Europe and North America [5]. Over 4 million migrants from Latin America have been estimated to reside in Europe, and the great majority of recent migration flows from the area have been directed to southern Europe (particularly Spain, Italy, and Portugal) [6,7].

The global epidemiology of CD has changed markedly in the last decades, and an improved knowledge of the difficulties involved in the diagnosis and management of these patients may contribute to improvements in control.

## 2. Epidemiology

Due to population mobility, urbanization, migration, and climate change, in recent decades, the epidemiological pattern of the disease has changed from a rural to a mostly urban pattern [1,8]. In the last few years, national control programs established by endemic countries have led to a reduction in prevalence in these areas; however, a marked increase in the number of CD cases in non-endemic countries has been observed [1,8].

In endemic areas, vector-borne transmission is the most common mode of acquisition of the infection. Outside endemic areas, there have been rare reports of local transmission in the United States, mostly based on investigations of blood donors with positive screening results [9]. Vector-borne transmission of CD has not been described in Europe. However, modelling of climatic suitability of CD vectors, has suggested that two triatomine species (*T. infestans, T. sordida*) could adapt to Southern European climate [10]; and there has been a report of vector *Triatoma rubrofasciata* being accidentally imported into Europe [11], highlighting the need for international vector surveillance to prevent future vector-borne transmission of CD in the area. Oral transmission via contaminated water and/or uncooked food can cause sporadic outbreaks in endemic areas [12]. Other routes of infection can also occur in non-endemic areas. Vertical transmission occurs in around 5% of infants born from infected pregnant women and its risk is associated with a higher maternal parasitic load [13,14]. *Trypanosoma cruzi* can be transmitted via solid organ or bone marrow transplant and via blood transfusion and the risk varies depending on the blood component and/or type of organ transplanted. *T. cruzi* screening of the United States blood supply became widespread in 2007 and was nationwide by 2012 after the Food and Drug Administration (FDA) issued final guidance on recommended donor screening [15]. In Europe, only a minority of countries currently conduct systematic screening for *T. cruzi* infection in blood donors [16,17].

In European countries, CD is not systematically monitored, although the number of cases reported has increased in the last decade [16,18,19]. It is estimated that more than 4.6 million migrants from endemic countries live regularly in Europe, 2.2 million live in Spain, followed by Italy, UK, The Netherlands, France, Germany, Portugal and Switzerland [7]. A study performed in 2009 calculated that 1.8 to 2.8% of Latin American migrants in Europe had been infected with *T. cruzi* with or without chronic disease [3]. In the same study, Spain was the country with the highest seroprevalence of infected migrants (2.3% and 3.8%) followed by Belgium (1.6–2.1%) and Italy (1.6–2%) [3].

The majority of data on CD in Europe come from Spain, which has been the main destination for Latin American migration to Europe in the last decade [16,20]. In a recent study, the estimated seroprevalence among Latin American migrants (2010–2018) was 2.1%, with Bolivian migrants representing more than half of cases [2]. A systematic review, including previous studies, calculated a total pooled seroprevalence of *T. cruzi* infection in Latin American migrants in Spain of 6.1% (95% CI: 3.2–9.7%; I2 = 98.8%). Bolivia was the most represented country of origin (90%), followed by Argentina (3.3%), Paraguay (2.5%) and Ecuador (1.2%) [21]. According to these more recent results, a general trend in decreasing seroprevalence in specific non-endemic areas in Europe may be occurring, possibly due among other factors, to better control strategies in the countries of origin.

The estimated seroprevalence in non-endemic areas varied substantially by country of origin. Another systematic review estimated that migrants to Europe from Bolivia had the highest seroprevalence of CD (18.1%, 95% CI: 13.9–22.7), followed by those from Paraguay (5.5%, 95% CI: 3.5–7.9). As for those born in Central American countries, El Salvador (5.6%, 95% CI: 1.6–11.7), Nicaragua (4.57%, 95% CI: 0.8–11.3), and Honduras (3.7%, 95% CI: 1.3–7.4) showed the highest seroprevalence in this area [22].

In Italy, a seroprevalence study performed between 2013 and 2020 showed a seroprevalence of CD of 16% among Latin American migrants; however, for individuals born after 1991 and 2011, this seroprevalence was only 2%, in line with seroprevalence trends from other European countries. [23]. A review of seroprevalence studies performed in Italy showed that there are great variations by region and country of origin, with seroprevalence data ranging from 1.3% to 17% and these could be as high as 30% when focusing on the Bolivian community only [24].

Germany has identified 81 cases of CD among more than 5000 people tested between 2000 and 2018 (this includes individuals where data on country of origin were not available or interpretable), Bolivia was again the most represented country of origin with more than half of the cases coming from this country [25].

A study performed in the UK reviewed all cases diagnosed in the country from 1995 and 2018. Sixty CD cases were identified, with 75% of them coming from Bolivia [18].

A review of recent CD prevalence data in Europe therefore reflects that CD remains a neglected tropical disease. There is a lack of robust studies, with scarcity of data, which are often outdated and appear to originate from reference and specialized centers mainly, representing significant gaps in knowledge [4].

## 3. *T. cruzi* Infection in Pregnancy and Vertical Transmission

Approximately 1.12 million women of childbearing age are estimated to be infected worldwide [26], and as neither mothers nor newborns are routinely screened for CD in many endemic and non-endemic areas, this figure likely represents a large underestimate [27].

Mother-to-child transmission of CD is considered a public health problem and is now the main mode of infection in non-endemic countries, but also in many regions of endemic countries where other ways of transmission have been controlled through intervention programs [28,29].

Chronic asymptomatic CD does not seem to affect fertility although further studies are needed to define the overall risk of infertility [30]. In mothers with chronic infections, several studies suggest that this condition does not influence the outcome of pregnancy or the health of newborns as long as there is no vertical transmission to the fetus [30,31,32]. In situations of acute maternal infection or vertical transmission, an increased risk of prematurity, low birth weight and premature rupture of membranes due to inflammatory involvement of the placenta, as well as the risk of polyhydramnios have been reported [30,33].

The risk of vertical transmission from a pregnant woman with CD is estimated to be about 5% (up to 10% in high-risk endemic areas) [34], and the number of *T. cruzi*-infected newborns in Latin America has been calculated to be around 9000 each year [13,33]. Outside endemic countries, the rate of vertical transmission of *T. cruzi* infection is estimated at around 3.8%, according to a recently published meta-analysis [35].

Symptomatic congenital CD occurs in nearly 30% of cases and is associated with a mortality of around 2% [36]. Mother-to-child infection can occur at any stage of the disease, acute or chronic, and appears to depend on interactions between the mother, placenta, fetus, and parasite [33]. Although the mechanism of vertical transmission of *T. cruzi* is not well understood, high parasite burden in the pregnant woman has been found to be a risk factor [14]. Measurement of IgG subclasses has been studied and may identify women at higher risk of vertical transmission [37]. The role of other factors such as composition of the maternal microbiota has not yet been explored and investment in this area could open new research paths [33].

Previous treatment has been shown to clearly reduce the risk of vertical transmission [38,39]. As available antiparasitic drugs for CD should not be offered during pregnancy, screening prior to pregnancy is essential. Feasibility studies of an experimental therapeutic vaccine against Chagas disease have also been proposed, especially to control and prevent vertical transmission [39,40].

A total of 783,871 women of childbearing age from Chagas-endemic areas were estimated to be living in Spain in 2018, and up to 23,400 (3.0%) could be infected with *T. cruzi* [2]. Two screening studies preformed in Italy in pregnant women showed great variation in CD seroprevalence ranging from 0.6 to 8.7% [41,42].. Some of the main reasons for the unknown incidence in most European countries are the low awareness of CD as a public health problem and the lack of standardized protocols for diagnosis, whilst some countries such as Spain have national guidance [21]; CD screening was not part of the recommendations on screening for infectious diseases in newly arrived migrants to EU/EEA issued by European Centre for Disease Prevention and Control (ECDC) in 2018 [19,43]. In 2002, the WHO published the first criteria for CD diagnosis recommending the screening of both at-risk pregnant women and their newborns [44]. Following this, other international strategies and action plans, such as PAHO’s Elimination of Mother-to-Child Transmission (EMTCT) initiative, WHO’s Roadmap for NTDs, and the CUIDA project, have been launched to control the burden of congenital CD [1,26,27,45]. Most of these have been implemented in endemic areas. In Europe, there is no uniform legislation for screening of pregnant women at risk and follow-up of their offspring [6,46]. As only general recommendations are widely given, there is little consensus between ministries or regional health departments where different protocols apply [6].

Countries such as Spain, Italy, and Switzerland have implemented screening programs for congenital CD at the national level, but only four European regions (three in Spain and one in Italy) have a specific official algorithm for diagnosis [6].

Several antenatal screening programs have been evaluated with favorable results but there is still a need to improve interventions to reach more of the target population via a multidisciplinary approach [2,47,48]. The recent study from Spain highlighting the high rates of underdiagnosis and undertreatment emphasizes the need for novel and more efficient strategies [2].

Standardized screening for CD of all fertile or pregnant women from endemic countries or whose mothers were born in endemic countries is essential to control transmission and prevent new cases. The integration of CD diagnosis, treatment, and care plans into health services can also contribute to the elimination of congenital transmission. The involvement of European countries in the applicability of this screening and in the visibility of this problem is essential to ensure its success.

## 4. Pediatric Chagas Disease

WHO data provide estimates on the number of babies born annually with CD in Latin America [49], but there are no official specific data on the number of pediatric cases worldwide.

Information on the prevalence of pediatric cases of CD outside endemic countries is scarce. In a systematic review and meta-analysis calculating the prevalence of CD in Latin American migrants living in Europe, all the included studies reported only cases in adults [22].

A recent study conducted in Spain reported an estimated 55,367 migrants with CD in 2018, of whom 613 (1.1%) were children under 14 years of age. This resulted in an estimated prevalence of Chagas disease among individuals from *T. cruzi*-endemic countries of 2.6% in adults and 0.1% in children [2].

Few series based on pediatric cases of CD in Europe have been published. The largest included 51 cases of CD in children, 50.9% of whom were born in Spain and all of whom had Latin American mothers [50]. Another study reported 45 pediatric *T. cruzi* cases diagnosed in Spain and Switzerland and all were children of Latin American origin. Two children were diagnosed during the acute phase at birth and the remaining 43 were in the chronic phase of the infection [51]. Another Spanish study screened 157 children born in Latin America and 45 born in Spain whose mothers were of Latin American origin and found a prevalence of CD of 10.8% (17 cases) and 11% (5 cases), respectively. PCR for *T. cruzi* was performed in 20 of the 22 cases diagnosed by serological screening and was positive in 12 cases (60%) [52]. For children born in Latin America, Bolivia was the most frequent area of origin [50,51,52].

Pediatric cases have been reported to be asymptomatic in 60–90% of cases [50,51,52]. If left untreated, most children enter a prolonged asymptomatic chronic phase in which about one third of patients may develop late complications [53]. Benznidazole is the treatment of choice for children, with nifurtimox being the only other available option [54,55]. Benznidazole achieves a cure rate of up to 100% in children with congenital infection treated in the first year of life and 76% in patients with acute infection, with cure rates decreasing to 9–15% in adult patients with chronic disease [56]. Nifurtimox has cure rates of 86% in children and 7–8% in adults [56]. Therefore, the efficacy in children is much higher than in adults and the earlier the treatment is administered, the better the response. Published data have reported cure rates of 80% in children aged less than 1 year compared to 4.3% in older children [50]. Moreover, children show better tolerance to treatment with fewer adverse effects than adults [57], especially when treatment is administered in patients younger than 7 years of age [50].

Guidelines recommend screening for CD in pregnant women from endemic countries, in children born to infected mothers (ideally at birth) and in all children from endemic areas, regardless of age [21,49,54,55]. Screening during pregnancy and at birth is increasingly being considered in clinical practice. However, systematic screening at all pediatric ages appears to be neglected. Children with CD are particularly vulnerable, not only because of the pathogenic potential of the infection, but also because of their often challenging socio-economic situations, including migration and international adoption [58].

Given the current migration dynamics and the indolent course of the disease during the initial decades after infection, it can be presumed that a substantial number of older children and teenagers living in Europe have undetected *T. cruzi* infection. There is a need for increased public health and clinical attention to CD for children living in Europe. Epidemiological surveillance should be reinforced to determine the true prevalence of pediatric CD in the region. In addition, the development of appropriate screening and early treatment interventions would increase cure rates and prevent long-term complications.

## 5. Transfusion and Transplant-Associated *T. cruzi* Infections

Blood transfusion represents another mode of transmission for *T. cruzi*, both in endemic and non-endemic areas [59]. It has been estimated that 300 to 800 cases of transfusion-transmitted CD have occurred in the last decades globally [60,61]. In Europe, cases of transfusion-transmitted CD have been reported in Spain [17,62,63,64].

Several factors impact on the risk of CD transmission via blood transfusion including the prevalence of the disease in the area and the quantity and type of blood product used (platelets are the blood product with the highest transmission rate whilst no transmission has been reported from plasma derivatives) [65]. Parasitemia and strain of the parasite, and the immune status of the recipient also play a role in transmission risk. Prevention strategies such as deferral of donors at risk for CD and donor and/or donation screening (negative test in a validated antibody test) prior to transfusion are key for reducing the risk of transfusion transmission as, often, donors affected by CD are asymptomatic and unaware of the disease [17,66,67]. Selective *T. cruzi* screening identifies donors at risk of CD and subsequently tests them for *T. cruzi*. This method has been shown to be nearly as effective as universal screening, but at a reduced cost [68].

Current European directives 2004/33/CE on technical requirements for blood and blood components state that patients affected or who have been affected by CD should be excluded permanently from blood donation programs [69]. This policy does not consider those donors who have recently been exposed to CD or have yet to be screened. Post-travel deferral recommendations that might be adequate for viral infections contracted whilst travelling do not apply to CD, which is often asymptomatic and chronic [46]. Moreover, donor deferral has not been shown to be completely effective [66], and contributes to a loss of donors.

A study of CD and blood transfusion in non-endemic countries [17] showed that there is great heterogeneity in Europe in terms of transfusion risk but also that different countries have adopted different preventive strategies to avoid transfusion transmission not necessarily in line with the European directives. Spain, France, and the United Kingdom conduct systematic screening for *T. cruzi* infection in blood donors, whilst Belgium, Germany and Italy use pre-transfusion questionnaires [16].

The seroprevalence of CD among blood donors has a high variability between countries and regions with studies screening high-risk donors ranging from no detected cases to as high as a 3.9% seropositivity in an Italian study [64,70,71,72,73,74,75]. The asymptomatic and chronic nature of CD, together with the lack of awareness of patients and health care professionals, might contribute to transfusion transmitted cases being undetected [9].

*T. cruzi* infection also poses a challenge in transplant medicine. Solid organ transplant recipients infected with CD are at risk of reactivation. Reactivation is greater in CD patients receiving a heart transplant (50%) and up to 20% in the context of renal transplantation, whereas reactivation in liver transplant recipients has not been well described. The risk is highest during the first year post-transplant and varies depending on the degree of immunosuppression. Receiving anti-parasitic treatment for *T. cruzi* prior to transplantation for transplant recipients suffering from CD does not necessarily reduce the risk of reactivation but might be considered on an individual basis [76,77]. Reactivation of CD has also been reported in bone marrow transplants, monitoring of CD and consideration of preemptive therapy might be considered [78,79,80].

The risk of transmission from a *T. cruzi*-infected donor depends on the organ transplanted. The risk for liver and kidney transplant ranges from 10 to 20%, and reaches 75% for heart transplants [77,81,82,83].

EU directive 2006/17 on technical requirements for the donation, procurement and testing of human tissues and cells recommends that donors at risk of CD based on history should be tested. France, Italy, Spain, and the United Kingdom have implemented measures to prevent transmission via organ, tissue, and cell transplantation [84].

The decision to use the organ from an infected donor may occasionally be considered and will depend on the degree of urgency for transplantation and the potential risk of the infection in the recipient, although some institutions and/or countries exclude these organs for donation. Use of organs from donors with acute infection and use of the heart/intestines from donors with chronic *T. cruzi* infection is contraindicated. In the case of infected living donors, specific anti-parasitic treatment before donation could be considered to reduce parasitic load and transmission [77,85,86]. There are currently not enough data to recommend the use of prophylaxis for the recipient, and regular monitoring should take place to detect transmission [77,87].

## 6. *T. cruzi* and Co-Infections

Migrants from *T. cruzi*-endemic areas may harbor latent infections produced by both cosmopolitan and geographically restricted pathogens. Vulnerable groups may be at greater risk for chronic infections such as HIV, hepatitis B and C virus infections, and strongyloidiasis. Studies reporting on screening for *T. cruzi* in HIV-positive patients in Europe have found a prevalence ranging from 0.5 up to 10%, depending in part on whether a single positive result or seropositivity for two seroassays were considered for diagnosis, as per WHO recommendations [88,89,90]. Serodiscordance for *T. cruzi* may be a potential barrier to diagnosis in immunocompromised patients, although studies comparing the performance of assays in these different groups of patients are scarce.

European studies testing for strongyloidiasis in patients with CD have found a seroprevalence of 10–15% [91,92,93]. The possible immunomodulatory role of helminth co-infections on CD has also been explored. A study performed in over 200 patients diagnosed with CD during blood donation in a region in Spain found a seroprevalence for *S. stercoralis* of 11% [93]. Positive *T. cruzi* RT-PCR was more frequent in patients with positive *Strongyloides* serology when compared to those with negative serology (56% vs. 33%). A further study evaluating the relationship between clinical, microbiological, and epidemiological characteristics of CD patients and the presence of helminth infections failed to demonstrate significant differences when clinical and epidemiological aspects were considered. However, the proportion of patients with positive *T. cruzi* RT-PCR was significantly higher among patients with helminth infections (mainly strongyloidiasis) compared with patients without these infections (75% vs. 36%) [94]. These findings warrant future investigations as chronic helminth infections may modulate the host immune system and may modify the response to other infections.

These effects may be especially relevant in the context of immunosuppressed patients and latent infections which may reactivate leading to severe disease such as strongyloidiasis. Opportunities may arise for researching the interaction between *T. cruzi* and other infections in regions where the possibility of re-exposure is extremely rare but co-infections also add to the complexity of managing patients with imported infections and other co-morbidities in a non-endemic area.

European Centre for Disease Prevention and Control (ECDC)’s public health guidelines for recently-arrived migrants consider screening for active and latent TB, HIV, HCV, HBV, strongyloidiasis and schistosomiasis, conditional to the burden of each disease in the countries of origin [19]. Latin American migrants screening for both *T. cruzi* and *Strongyloides* spp. infection should therefore be considered even in asymptomatic cases.

## 7. Screening Strategies and Mitigation of the Burden of Under-Diagnosis of Chagas Disease in Non-Endemic Areas

Screening strategies play a crucial role in the early detection and management of CD, as early intervention can prevent severe complications such as cardiomyopathy and digestive involvement. As mentioned previously, screening and treatment of women in childbearing age prevents mother to child transmission [14,95]. Although there are different local strategies and programs in the European countries with highest rates of Latin American migrants, there are considerable disparities between them, and only few centers/countries have established systematic monitoring of CD in pregnant women from Latin American countries [6,21,43].

An economic evaluation of the most efficient screening strategy among Latin American migrants living in Spain showed that screening of CD in this population is cost-effective [96]. One of the most efficient strategies from both perspectives was screening of pregnant women, their newborns, and first- and second- degree relatives of disease-positive mothers. From the Spanish National Health System perspective, including ‘relatives of a disease-positive mother’ would involve a mean increase of EUR 301 per patient and QALY gained [96]. A model comparing the impact of systematic screening (all asymptomatic Latin Americans seen at primary care settings, treatment, and follow-up of positive cases) with screening and treatment of symptomatic persons in Spain, proved to be a cost-effective strategy, even in scenarios with a very low disease prevalence (0.05%) [97]. As previously noted, screening strategies for potential blood and organ donors with an epidemiological risk of the infection should be strengthened in Europe.

Health system accessibility should also be considered. A recent qualitative study on diagnostic pathways in a European country identified administrative, physical and time-related potential problems in accessibility for individuals actively seeking testing and other factors such as low perception of risk and fear of stigma in individuals not actively seeking testing for CD [98]. Point-of-care testing may overcome some of the challenges faced when screening these high-risk vulnerable populations and may be used in community screening programs. Rapid diagnostic tests (RDTs) are an easy-to-use alternative to conventional tests with high sensitivity and specificity which can improve the access to diagnosis [99]. Innovative strategies such as coupling ELISA to smartphones for testing of chronic and congenital CD in primary health care or community settings have also been developed to facilitate early diagnosis and management [100]. Once a diagnosis is established, a strategy facilitating linkage to care would also be necessary.

## 8. Future Perspective

**Figure 1:**
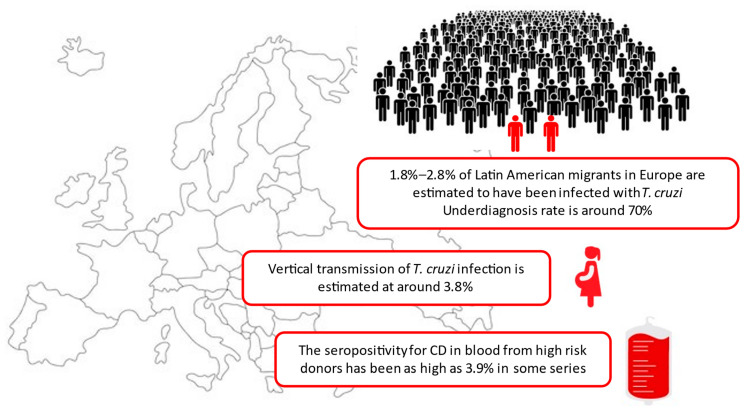
Schematic representation of Chagas disease burden in Europe [2,3,36,70].

The growing burden of Chagas disease in Europe currently represents both a reality and a challenge for policy makers (Figure 1). Most data available on CD in Europe are from Spain, as this is the second country with more Latin American migrants after the US. It must be highlighted that data on CD in Europe are scarce and often outdated bearing in mind the dynamic nature of migration. Figures which could be highlighted are the high indices of underdiagnosis and undertreatment of the disease and the lack of official standardized screening guidelines especially for vulnerable groups. in many non-endemic countries. Recent EU/EEA public health guidance on screening for infectious diseases in newly arrived migrants has failed to include CD. Due to its latent and chronic nature, *T. cruzi* infection remains an invisible disease in many areas and an effective call to action is necessary to implement measures which could have a significant impact. Integration of CD diagnosis, treatment and care plans into health services may contribute to the elimination of congenital transmission. Strict implementation of policies to regulate the safety of blood products and organs used for transplantation may ensure the complete control of this mode of transmission. As illustrated, the management of patients with Chagas disease, co-infections and co-morbidities in a non-endemic area may also pose a challenge for health care professionals. Key points related to current CD epidemiology are illustrated in Box 1. Administrative and time-related problems, low perception of risk and fear of stigma have all been identified as potential problems for individuals in the context of CD testing. Efforts should be made to increase awareness on the disease, obtain robust surveillance data in Europe, and deliver high-quality management to the detected cases. Possible solutions to overcome these challenges include community outreach programs, use of point-of-care validated tests and application of more novel smartphone-based digital technologies to enhance CD knowledge and screening, and facilitate linkage to care.

Box 1 **Main epidemiological features and challenges for the management of Chagas Disease in Europe.**

**Key points**

- Chagas Disease, no longer a geographically restricted infection, has become a global health challenge;
- Nearly 5 million American migrants live in Europe, an estimated 1.8%–2.8% of them infected with *T. cruzi, *and 0.1% are children;
- Main routes of transmission in non-endemic areas are transfusion transmitted CD and vertical transmission;
- The rate of vertical transmission of *T. cruzi* infection outside endemic countries is estimated at around 3.8%;
- The proportion of donated blood for high risk donors infected by *T. cruzi *may have great variability (highest rate reported in Europe 3.9%; Italy 2010);
- CD may be transmitted from infected donors to 10–20% of kidney and liver transplant recipients and 75% of heart transplant recipients;
- CD patients undergoing immunosuppression and receiving a transplant are at risk of disease re-activation;
- Surveillance is essential to understand the burden of CD in non-endemic countries and prevent ongoing transmission. Selective screening of people at risk of CD, especially women of childbearing age should be included in European Health Policies;
- People diagnosed with CD should be screened for other geographically restricted infections such as strongyloidiasis.
